# Sequential treatment of ADHD in mother and child (AIMAC study): importance of the treatment phases for intervention success in a randomized trial

**DOI:** 10.1186/s12888-018-1963-9

**Published:** 2018-12-13

**Authors:** Christopher Hautmann, Manfred Döpfner, Josepha Katzmann, Stephanie Schürmann, Tanja Wolff Metternich-Kaizman, Charlotte Jaite, Viola Kappel, Julia Geissler, Andreas Warnke, Christian Jacob, Klaus Hennighausen, Barbara Haack-Dees, Katja Schneider-Momm, Alexandra Philipsen, Swantje Matthies, Michael Rösler, Wolfgang Retz, Alexander von Gontard, Esther Sobanski, Barbara Alm, Sarah Hohmann, Alexander Häge, Luise Poustka, Michael Colla, Laura Gentschow, Christine M. Freitag, Katja Becker, Thomas Jans

**Affiliations:** 1 Department of Child and Adolescent Psychiatry, Psychosomatics and Psychotherapy, Medical Faculty of the University of Cologne, Cologne, Germany; 2 School of Child and Adolescent Cognitive Behavioral Therapy (AKiP), University Hospital Cologne, Pohligstraße 9, 50969 Cologne, Germany; 3 Department of Child and Adolescent Psychiatry and Psychotherapy, Charité – Universitätsmedizin Berlin, Freie Universität Berlin, Humboldt-Universität zu Berlin, and Berlin Institute of Health (BIH), Berlin, Germany; 4 Department of Child and Adolescent Psychiatry, Psychosomatics and Psychotherapy, Center for Mental Health, University Hospital of Würzburg, Würzburg, Germany; 5 Department of Psychiatry, Psychosomatics and Psychotherapy, Center for Mental Health, University Hospital of Würzburg, Würzburg, Germany; 6 Department of Psychiatry, Psychosomatics and Psychotherapy, Medius Clinic, Kirchheim, Germany; 7 Department of Child and Adolescent Psychiatry, Psychotherapy and Psychosomatics, Medical Faculty and Medical Center – University of Freiburg, Freiburg, Germany; 8 Department of Psychiatry and Psychotherapy, Medical Center – University of Freiburg, Faculty of Medicine, University of Freiburg, Freiburg, Germany; 9 Department of Psychiatry and Psychotherapy, University Hospital Bonn, Bonn, Germany; 10 Institute for Forensic Psychology and Psychiatry, Saarland University Faculty of Medicine, Homburg/Saar, Germany; 11 Department of Psychiatry and Psychotherapy, University Medical Center Mainz, Mainz, Germany; 12 Saarland University Hospital: Department of Child and Adolescent Psychiatry and Psychotherapy, Saarland University Faculty of Medicine, Homburg/Saar, Germany; 13 Clinic for Psychiatry and Psychotherapy, Central Institute of Mental Health Mannheim, Medical Faculty Mannheim, University of Heidelberg, Mannheim, Germany; 14 Department of Child and Adolescent Psychiatry and Psychotherapy, Johannes Gutenberg University Mainz, Mainz, Germany; 15 Department of Child and Adolescent Psychiatry and Psychotherapy, Central Institute of Mental Health, Medical Faculty Mannheim, University of Heidelberg, Mannheim, Germany; 16 Department of Child and Adolescent Psychiatry, University Medicine Göttingen, Göttingen, Germany; 17 Department of Child and Adolescent Psychiatry, Medical University of Vienna, Vienna, Austria; 18 Department of Psychiatry and Psychotherapy, Campus Benjamin Franklin, Charité – Universitätsmedizin Berlin, Berlin, Germany; 19 Department of Psychiatry, University Medical Center Rostock, Rostock, Germany; 20 Department of Child and Adolescent Psychiatry, Psychosomatics and Psychotherapy, Frankfurt University Hospital, Frankfurt, Germany; 21 Department of Child and Adolescent Psychiatry and Psychotherapy, Philipps University Marburg, Marburg, Germany

**Keywords:** Mothers, Children, Adult treatment, Parent training, Efficacy

## Abstract

**Background:**

The efficacy of parent-child training (PCT) regarding child symptoms may be reduced if the mother has attention-deficit/hyperactivity disorder (ADHD). The AIMAC study (ADHD in Mothers and Children) aimed to compensate for the deteriorating effect of parental psychopathology by treating the mother (Step 1) before the beginning of PCT (Step 2). This secondary analysis was particularly concerned with the *additional* effect of the Step 2 PCT on child symptoms after the Step 1 treatment.

**Methods:**

The analysis included 143 mothers and children (aged 6–12 years) both diagnosed with ADHD. The study design was a two-stage, two-arm parallel group trial (Step 1 treatment group [TG]: intensive treatment of the mother including psychotherapy and pharmacotherapy; Step 1 control group [CG]: supportive counseling only for mother; Step 2 TG and CG: PCT). Single- and multi-group analyses with piecewise linear latent growth curve models were applied to test for the effects of group and phase. Child symptoms (e.g., ADHD symptoms, disruptive behavior) were rated by three informants (blinded clinician, mother, teacher).

**Results:**

Children in the TG showed a stronger improvement of their disruptive behavior as rated by mothers than those in the CG during Step 1 (Step 1: TG vs. CG). In the CG, according to reports of the blinded clinician and the mother, the reduction of children’s disruptive behavior was stronger during Step 2 than during Step 1 (CG: Step 1 vs. Step 2). In the TG, improvement of child outcome did not differ across treatment steps (TG: Step 1 vs. Step 2).

**Conclusions:**

Intensive treatment of the mother including pharmacotherapy and psychotherapy may have small positive effects on the child’s disruptive behavior. PCT may be a valid treatment option for children with ADHD regarding disruptive behavior, even if mothers are not intensively treated beforehand.

**Trial registration:**

ISRCTN registry ISRCTN73911400. Registered 29 March 2007.

**Electronic supplementary material:**

The online version of this article (10.1186/s12888-018-1963-9) contains supplementary material, which is available to authorized users.

## Background

The general efficacy of behavioral interventions for children with attention-deficit/hyperactivity disorder (ADHD), including parent training, has been comprehensively investigated [1–3]. However, as treatment success varies across families [4], several studies investigated the moderating role of parental ADHD in the efficacy of behavioral parent training. This introduction builds in part on two comprehensive reviews summarizing the findings from this research area [5, 6].

The heritability of ADHD is high [7] and many children with ADHD have parents who show ADHD symptoms themselves [8, 9]. Furthermore, parents with ADHD are more likely to have deficits in parenting than parents without ADHD [10–12].

Most importantly, parental ADHD has been linked to reduced efficacy of behavioral parent training. Diminished effects have been reported for child ADHD symptoms and behavioral problems [13, 14] as well as for parenting [13, 15]. However, there are also studies reporting no deteriorating effect of parental ADHD on child benefit from treatment [13, 15, 16], which may at least partly be explained by less affected parents and more personalized treatment approaches in the respective studies [5].

For adults with ADHD, the current first-line treatment option is pharmacotherapy [17, 18]. Effect sizes of stimulants on ADHD symptoms are in the medium to large range [19, 20] and are comparable to those reported for children and adolescents [21]. Cognitive behavioral therapy has been less intensively investigated [18, 22], but the available evidence shows some positive findings [23–26].

Treatment of ADHD in adults (pharmacotherapy, cognitive behavioral therapy) may also affect parenting and child outcome. Only a small number of trials have investigated this issue with regard to pharmacotherapy. The current evidence is mixed [27–29] and it has been suggested that the effects of pharmacotherapy on parenting might be limited and insufficient [5]. To our knowledge, the effects of cognitive-behavioral interventions in adults have not yet been evaluated with respect to child outcomes.

Some studies tested the combined effect of parent ADHD treatment and behavioral parent training in terms of improving child outcome [30–32]. In such cases, the adult treatment might serve two different purposes: First, it may have a (limited) more or less direct effect on parenting and on child outcome (via parenting); and second, improvements in neurocognitive functioning might help parents to participate in and benefit from the training (e.g., sustaining attention during the sessions) and to fulfill accompanying requirements (e.g., homework).

In a study by Babinski et al. [30], the combination of pharmacotherapy for adults and behavioral parent training revealed no treatment advantage over behavioral parent training alone with regard to observed parenting and child behavior. However, parents only received stimulants before and after the parent training intervention period. Therefore, the study was able to provide information about the short-term effects of stimulants on parenting, but not about the potential treatment advantage of medication for training participation.

The AIMAC (ADHD in Mothers and Children) study tested the efficacy of combined mother and child ADHD treatment in a randomized controlled trial with an active control group [32–34]. In the first phase (Step 1), mothers of the treatment group (TG) received group psychotherapy plus stimulant medication, while mothers of the control group (CG) received supportive counseling only. In the second phase (Step 2), treatment of the mother was maintained and mothers of both groups were additionally provided with the same individual parent-child training. The results revealed that after Step 2, mothers of the TG showed a stronger ADHD symptom reduction, but no group differences were detected regarding children’s ADHD symptoms and disruptive behavior [32].

The current study constitutes a supplemental analysis of the AIMAC study by Jans et al. [32]. The previous main analysis focused on the comparison of the CG and TG (between-group analysis) concerning the combined effect of the mother and child treatment. As the main analysis revealed no group differences on important child domains, the present re-analysis aimed to establish the relative importance of the two treatment phases (Step 1, Step 2). For this purpose, the observed total change was split not only by group but also by treatment phase. Comparisons were conducted between groups (Step 1: CG vs. TG; Step 2: CG vs. TG) as well as within groups (CG: Step 1 vs. Step 2; TG: Step 1 vs. Step 2). The latter analyses served as a type of within-group control design (e.g., stronger child symptom reduction during Step 2 compared to Step 1 would have been considered as evidence for the efficacy of the parent-child training).

We were particularly interested in two research questions (primary testing approach in parentheses): (i) Is parent-child training useful for mothers with ADHD who have been minimally or intensively treated beforehand (CG: Step 1 vs. Step 2; TG: Step 1 vs. Step 2)? (ii) Do children benefit from intensive pharmacological and psychological treatment of the mother (Step 1: TG vs. CG)?

The first question concerned the efficacy and importance of the parent-child training in the treatment groups, a subject that is crucial for treatment planning and that is unresolved with regard to mothers with ADHD. On the one hand, for parents with ADHD, the efficacy of parent-child training has been called into question and is often considered to be reduced; on the other hand, non-significant findings have also been reported, and it has been suggested that for personalized parent training, the treatment effects might not be attenuated even in mothers with ADHD [5]. Furthermore, the main analysis of the AIMAC study did not reveal group differences on important child domains, despite the successful treatment of the mothers of the TG (e.g., ADHD symptoms, disruptive behavior) [32]. Contrary to our own previous hypotheses, we therefore assumed post hoc that the parent-child treatment actually did work in both treatment groups due to the individualized approach. Particularly for the CG, where the mother’s treatment was of low intensity, we expected the within-group control comparison to be significant, meaning that we would find a stronger symptom reduction during the Step 2 parent-child training than during the Step 1 treatment of the mother (CG: Step 1 vs. Step 2). For the TG, we also considered the parent-child training to be effective. However, regarding the within-group comparison (TG: Step 1 vs. Step 2), clear hypotheses were more difficult to derive for the TG, as here, the Step 1 treatment of the mother was more intense and at least for pharmacotherapy in adults, (limited) effects on parenting and child symptoms have been reported [27, 29]. We thus considered it more likely that a more homogenous and less distinct pattern of change would be observed across the treatment phases for the TG (TG: Step 1 vs. Step 2) and therefore, if additional effects were to be observed, we expected them to be rather small.

The second research question concerned the effects of the pharmacotherapy and psychological treatment of the mothers in the TG on the child’s symptoms when compared with supportive counseling for the mother (Step 1: TG vs. CG). ADHD pharmacotherapy for adults has shown some positive indirect effects on their children, but the sustainability of the impact has been questioned [5]. To our knowledge, the effect of adult psychotherapy on children has not been investigated so far, and nor has the combination of pharmacotherapy and psychotherapy been tested. We consider this to be the first trial to investigate this subject using a randomized design (Step 1: TG vs. CG). Due to the indirect effect of the treatment on the child, we expected to find rather small effects in favor of the TG.

## Methods

### Design

The design consisted of a blinded randomized multicenter parallel-group trial with two treatment arms (TG, CG). Families were allocated to the treatment arms by block randomization at a ratio of 1:1 and were stratified by center.

### Sites

The trial was conducted at five specialized units of university hospitals in Germany (Berlin, Freiburg, Homburg, Mannheim, Würzburg) and was coordinated by the University Hospital of Würzburg. Families were primarily recruited from patients who were referred to the hospitals but were also recruited via local child psychiatrists and newspaper and website advertising.

### Inclusion and exclusion criteria

Inclusion and exclusion criteria concerned mothers and their children and have been reported in detail elsewhere [32–34]. Mothers had to fulfill the following inclusion criteria: (a) age 18 to 60 years; (b) diagnosis of ADHD according to the *Diagnostic and Statistical Manual of Mental Disorders* (4th ed.; DSM-IV) [35]; (c) a score ≥ 30 on the Wender Utah Rating Scale, German short version (WURS-K) [36]. Exclusion criteria for mothers were: (a) IQ < 85; (b) psychotherapy for ADHD, methylphenidate treatment or parent-child training within the last 6 months before baseline assessment; (c) current psychotherapeutic or psychopharmacological treatment, (d) methylphenidate intolerance; (e) indication for inpatient treatment; (f) insufficient German language skills; (g) severe comorbid psychological disorder (e.g., schizophrenia, bipolar disorder); (h) medical contraindication (e.g., seizures, thyroid function, pregnancy).

Child inclusion criteria were: (a) age 6 to 12 years; (b) diagnosis of ADHD according to DSM-IV criteria; (c) no medication or stable medication for at least 4 weeks before baseline assessment. Exclusion criteria were: (a) IQ ≤ 80; (b) indication for inpatient treatment; (c) insufficient German language skills; (d) severe comorbid psychological disorder (e.g., pervasive developmental disorder, psychosis).

### Sample

The AIMAC study sample has been described in detail elsewhere [32, 34]. Briefly, during the trial, 444 families were contacted and pre-screened, 206 were assessed for eligibility, and 144 families were randomized. The current analysis is based on the full analysis set (FAS), considering only families with the primary outcome (child’s combined ADHD-ODD score) available at baseline (*n* = 143) [32].

Across the treatment groups, mothers were on average 38.30 (*SD* = 5.70) years old. Regarding DSM-IV ADHD diagnoses, 65.7% showed the combined type, 23.8% the predominantly inattentive type and 10.5% the predominantly hyperactive-impulsive type. At least one current or past comorbid disorder was detected in 71.3% of the mothers, with a single episode of major depressive disorder (26.6%) and recurrent major depressive disorder (21.7%) being the most prevalent conditions. Prior to the start of the study, 55.9% of the mothers had already received psychiatric or psychotherapeutic treatment and 14.0% had been previously treated with some stimulant medication (methylphenidate, amphetamine, other) and 0.7% had been prescribed atomoxetine.

In the FAS sample, children were predominantly male (73.4%) and were on average 9.44 (*SD* = 1.71) years old. With respect to the DSM-IV diagnosis of ADHD, 52.4% of the children showed the combined type, 39.2% the predominantly inattentive type and 8.4% the predominantly hyperactive-impulsive type. At least one current comorbid disorder was apparent in about half of the children (47.6%), with oppositional defiant disorder (ODD) being the most prevalent condition (30.1%). Prior to the start of the trial, 81.1% of the children had received psychiatric or psychological treatment. Furthermore, 53.8% had previously received stimulant medication (methylphenidate, amphetamine, other) and 2.8% had been treated with atomoxetine. During the trial, 74.8% of the children received ongoing psychopharmacological medication that had been prescribed before the start of the study and was to be kept stable.

### Intervention

#### Intervention steps

The intervention comprised three steps in both intervention arms (Step 1, Step 2, Step 3; see Additional file 1: Figure S1). Step 1 served for the treatment of the mothers and lasted for 3 months. Mothers of the TG received multimodal treatment for adults with ADHD (group psychotherapy, psychopharmacotherapy) while mothers of the CG received supportive counseling. In Step 2, the treatment of the mothers was continued and the parent-child training began. The second period also lasted for 3 months, and the treatment protocol of the parent-child training was identical for both the TG and CG. Step 3 lasted for 6 months and served as a maintenance period for mother and child treatment.

Treatment for mothers was on a weekly basis (12 sessions) during Step 1 and every 4 weeks thereafter (10 sessions during Step 2 and Step 3). Parent-child training during Step 2 was scheduled every week (12 sessions) and two booster sessions were offered thereafter (Step 3). For reasons of simplicity, in the following, the Step 1 treatment phase is called mother treatment and the Step 2 treatment phase is called parent-child training.

#### ADHD treatment for mothers – TG

Group psychotherapy for mothers of the TG was based on a treatment manual founded in cognitive behavioral therapy and dialectical behavior therapy [25, 37]. Topics of the sessions are psychoeducation, mindfulness training, organizational skills, self-management, emotional regulation, impulse control, stress management and interpersonal problems. Each session was planned to last for 2 h, with groups comprising six to nine parents, and to include homework. Based on the needs of each patient, up to three individual sessions were offered.

Mothers of the TG additionally received pharmacological treatment with long-acting methylphenidate (Medikinet® retard) [38]. Therapy was started with 10 mg/d and was individually adjusted to daily dosages of up to 1.3 mg/kg. Multiple dosages per day were allowed.

#### ADHD treatment for mothers – CG

Mothers of the CG received individual supportive counseling. Sessions lasted for 15 to 20 min and topics were based on the individual needs of the mothers. Counseling was conducted in a non-directive manner and mothers were encouraged to find solutions to their problems on their own.

#### ADHD treatment for children

Parent-child training was based on the German treatment manual THOP (Therapieprogramm für Kinder mit hyperkinetischem und oppositionellem Problemverhalten [Therapy program for children with hyperkinetic and oppositional problem behavior]) [39] for children with hyperkinetic and oppositional problem behavior, which has been shown to be effective in the short term and have lasting effects in the long term [40–42]. The treatment included the following topics: developmental model of behavioral problems, identification of most concerning child problems, enhancing positive parent-child interaction, rules, effective commands, positive consequences and negative consequences, time-out and token economy [40]. The individual training was held in 1-h sessions and focused primarily on mothers and their children; however, fathers and teachers were included whenever needed and feasible.

### Assessment and informants

Assessments were conducted at baseline (T1), immediately after Step 1 about 3 months from baseline (T2), after Step 2 about 6 months from baseline (T3), and after the Step 3 maintenance period about 12 months from baseline (T4). A follow-up assessment took place after a maintenance period of approximately 1 year (T5).

The current analysis concerned the assessments T1 to T3, which spanned Step 1 (T1–T2) and Step 2 (T2–T3). Other measurement occasions were disregarded as not all outcome measures of analysis were collected at all assessment points.

The analysis included the ratings of three informants (blinded clinician, mother, teacher). The blinded clinician was not involved in the treatment and conducted clinical interviews with the mother and the child. Her or his rating was blind to the treatment condition, but given the nature of interviews with families, blindness to the time of the assessment may not have been ensured in all cases.

### Outcome measures

#### Assessment of child symptoms

The selection of instruments considered for this analysis concerned child externalizing behavior and its impact on the family, including reports of all three informants (blinded clinician, mother, teacher).

#### Kiddie-Sads-Present and Lifetime Version (K-SADS)

The K-SADS is a semi-structured interview developed to assess psychopathology in children aged 6 to 18 years [43, 44]. It was conducted by the blinded clinician, who interviewed mothers and children separately regarding the child’s behavior during the past 2 weeks. Besides categorical diagnostics, the interview can also be used for dimensional assessment [45]. The three scales Inattention (Inattention; nine items), Hyperactivity/Impulsivity (Hyp/Imp; nine items) and Oppositional Defiant Disorder (ODD; eight items) are reported.

#### Strengths and Difficulties Questionnaire (SDQ)

The SDQ is a questionnaire to assess behaviors, emotions and relationships in children [46]. The version for parents and teachers of children aged 4 to 17 years was used and was rated by both informants. For this analysis, the scales Hyperactivity (Hyperactivity; five items), Conduct Problems (Conduct; five items) and Emotional Symptoms (Emotional; five items) are reported.

#### Home Situation Questionnaire (HSQ)

The HSQ is a questionnaire to assess externalizing behavior of children in specific situations [47, 48]. Items were rated by mothers, and the total scale (Total; 16 items) was used.

#### Family Impact Questionnaire (FIQ)

The FIQ is a questionnaire to measure the impact of child externalizing behavior on family functioning [49]. Results of three scales Impact on Social Life (Social; 11 items), Positive Feelings Toward Child (Positive; seven items) and Negative Feelings Toward Child (Negative; nine items) based on ratings of the mothers are reported.

#### Assessment of maternal symptoms

The instruments concerned the ADHD symptoms of the mothers. Reports of the blinded clinician and the mothers were considered.

#### Conners’ Adult ADHD Rating Scales–Observer: Long Version (CAARS–O)

The CAARS–O are designed to assess ADHD symptom domains in adults rated by significant others (e.g., relatives, professionals) [50]. For the study, the questionnaire was completed by the blinded clinician. Results of the subscales Inattention and Memory Problems (Inattention; 12 items), Hyperactivity/Restlessness (Hyperactivity; 12 items) and Impulsivity/Emotional Lability (Impulsivity; 12 items) are reported.

#### Conners’ Adult ADHD Rating Scales–Self-Report: Long Version (CAARS–S)

The CAARS–S were developed analogously to the CAARS–O [50] and were rated by the mothers.

### Statistical analysis

All analyses were performed using the statistical software R (Version 3.4) [51], and for structural equation modeling in particular, the specific R package lavaan was used (Version 0.5) [52]. Change over time was investigated by piecewise latent growth models [53, 54]. The TG and CG were first analyzed separately (single-group analysis) and then jointly (multi-group analysis; for a graphical representation, see Additional file 1: Figure S2). To describe the growth process, three latent variables were considered for each treatment group, one random intercept and two fixed slope factors. The means of the first fixed slope factor (CG: ${\upalpha}_2^{\left(\mathrm{CG}\right)}$; TG: ${\upalpha}_2^{\left(\mathrm{TG}\right)}$) represented the change during the Step 1 treatment of the mother in each of the treatment groups (T1 to T2), and the means of the second fixed slope factor (CG: ${\upalpha}_3^{\left(\mathrm{CG}\right)}$; TG: ${\upalpha}_3^{\left(\mathrm{TG}\right)}$) indicated the change during the Step 2 parent-child training (T2 to T3). The mean of the random intercept factor (CG: ${\upalpha}_1^{\left(\mathrm{CG}\right)}$; TG: ${\upalpha}_1^{\left(\mathrm{TG}\right)}$) represented the average outcome at baseline (T1).

During the modeling process, a series of nested models were analyzed to test for differences among factor means (null hypotheses [H_0_] in parentheses). The first step of the analysis (single-group analysis) focused on the comparison of the Step 1 change with the Step 2 change in the respective treatment groups (within-group analysis; CG H_0_: ${\upalpha}_2^{\left(\mathrm{CG}\right)}$ = ${\upalpha}_3^{\left(\mathrm{CG}\right)}$; TG H_0_: ${\upalpha}_2^{\left(\mathrm{TG}\right)}$ = ${\upalpha}_3^{\left(\mathrm{TG}\right)}$). For each treatment group, two models were analyzed and compared by a Chi-square difference test, one with the means of the two slope factors freely estimated and one with the means constrained to be equal. Equal growth rates would indicate that the observed change during the Step 1 treatment of the mother and the Step 2 parent-child training did not significantly differ and consequently, that no additional effect of the parent-child training could be shown in a particular treatment group.

In the next step of the examination, the treatment groups were tested jointly (multi-group analysis) in order to conduct comparisons among them (between-group analysis). The analysis was based on the final models of the former single-group analyses. The purpose of the between-group analysis was to compare the change rates in the CG and TG during the Step 1 treatment of the mother (H_0_: ${\upalpha}_2^{\left(\mathrm{CG}\right)}$ = ${\upalpha}_2^{\left(\mathrm{TG}\right)}$) as well as to compare the change rates among the treatment groups during the Step 2 parent-child training (H_0_: ${\upalpha}_3^{\left(\mathrm{CG}\right)}$ = ${\upalpha}_3^{\left(\mathrm{TG}\right)}$). The Step 1 comparison was of particular interest, because it served as a test for the efficacy of the intensive treatment of the mother in the TG relative to the CG. The analytical procedure was analogous to the within-group comparison; models with freely estimated means and models with the means constrained to be equal were estimated and were compared by Chi-square difference test. In this case, equal growth rates would show that the change during a respective treatment period was comparable between the groups. The between-group analysis additionally included a comparison of the means of the intercept factors in the CG and TG (H_0_: ${\upalpha}_1^{\left(\mathrm{CG}\right)}$ = ${\upalpha}_1^{\left(\mathrm{TG}\right)}$), which served as a test for differences at the beginning of the treatment (T1).

Results for standardized variables as well as unstandardized variables (see Additional file 1) are reported. For standardization, a z-transformation was conducted with the grand mean and the grand standard deviation over time and group of all available data [55]. The transformation was conducted to increase the interpretability of the coefficients. Given this type of standardization and the fact that the means of the two slope factors represented the average change during Step 1 and Step 2, we interpreted them like a Cohen’s *d* effect size measure.

Model fit was considered satisfactory when, for the Chi-square (χ^2^) test, *p* > .05 and the comparative fit index (*CFI*) > .90. The root mean square error of approximation (*RMSEA*) was not used, because all models had small degrees of freedom and, moreover, had a small sample size [56]. Missing data were handled by full-information maximum likelihood [57]. For a person to be considered for analysis, at least one of the three assessment points (T1–T3) had to be available.

## Results

Descriptive statistics of the CG and TG for outcome measures before and after Step 1 and Step 2 treatment are reported in Additional file 1: Table S1. Depending on the outcome measure, 57–66 families from the CG and 65–77 families from the TG were included.

Results of the final latent growth curve models for standardized variables are provided for child outcome (Table 1) and mother outcome (Table 2). Reported are the means of the latent slope factors for the CG $\Big({\upalpha}_2^{\left(\mathrm{CG}\right)},{\upalpha}_3^{\left(\mathrm{CG}\right)}$) and TG $\Big({\upalpha}_2^{\left(\mathrm{TG}\right)},{\upalpha}_3^{\left(\mathrm{TG}\right)}$), which represent change in groups during Step 1 and Step 2. The results concern the final models after constraints among the means of the latent factors had been tested in the within- and the between-group analysis. All coefficients with an asterisk (^*^) indicate that the means are significantly different from zero. Further, coefficients of equal size indicate equality constraints among the parameters and show that the parameters did not significantly differ across time or group. For example, for K-SADS ODD (Table 1), change rates across groups and periods were all considered to be equal except for the CG during the Step 2 treatment (${\upalpha}_2^{\left(\mathrm{CG}\right)}$ = ${\upalpha}_2^{\left(\mathrm{TG}\right)}$ = ${\upalpha}_3^{\left(\mathrm{TG}\right)}$ = − 0.16; ${\upalpha}_3^{\left(\mathrm{CG}\right)}$ = − 0.53). Although the within- and between-group analyses were integrated, for reasons of convenience, the results are described separately in the following.

**Table 1:** Change in Child Outcome Variables During Step 1 Treatment of the Mother and Step 2 Parent-Child Training for Standardized Variables

Outcome & informant	Control group		Treatment group	
	${\upalpha}_2^{\left(\mathrm{CG}\right)}$	${\upalpha}_3^{\left(\mathrm{CG}\right)}$	${\upalpha}_2^{\left(\mathrm{TG}\right)}$	${\upalpha}_3^{\left(\mathrm{TG}\right)}$
K-SADS blinded clinician				
Inattention	−0.42^*^	− 0.42^*^	− 0.42^*^	− 0.42^*^
Hyp/Imp	− 0.25^*^	− 0.25^*^	− 0.25^*^	− 0.25^*^
ODD	− 0.16^*^	− 0.53^*^	− 0.16^*^	− 0.16^*^
SDQ mother				
Hyperactivity	−0.30^*^	− 0.30^*^	− 0.30^*^	− 0.30^*^
Conduct	− 0.05	−0.24^*^	− 0.24^*^	− 0.24^*^
Emotional	− 0.19^*^	− 0.19^*^	− 0.19^*^	− 0.19^*^
HSQ mother				
Total	−0.27^*^	− 0.27^*^	− 0.27^*^	− 0.27^*^
FIQ mother				
Social	−0.14^*^	− 0.14^*^	− 0.14^*^	− 0.14^*^
Negative	− 0.20^*^	− 0.20^*^	− 0.20^*^	− 0.20^*^
Positive	0.04	0.04	0.04	0.04
SDQ teacher				
Hyperactivity	−0.11^*^	− 0.11^*^	− 0.11^*^	− 0.11^*^
Conduct	− 0.17^*^	− 0.17^*^	− 0.17^*^	− 0.17^*^
Emotional	− 0.13^*^	− 0.13^*^	− 0.13^*^	− 0.13^*^

Note. Results concern the final model of a series of nested piecewise linear latent growth models (within- and between-group analysis). In the case of equal coefficients (i.e., α_2_ and α_3_ in TG and CG), change rates did not significantly differ across treatment groups and/or steps and were constrained to be equal. ${\upalpha}_2^{\left(\mathrm{CG}\right)}$ = mean of latent slope factor representing average change in CG during Step 1 (T1 to T2); ${\upalpha}_3^{\left(\mathrm{CG}\right)}$ = mean of latent slope factor indicating average change in CG during Step 2 (T2 to T3); ${\upalpha}_2^{\left(\mathrm{TG}\right)}$ = mean of latent slope factor representing average change in TG during Step 1 (T1 to T2); ${\upalpha}_3^{\left(\mathrm{TG}\right)}$ = mean of latent slope factor indicating average change in TG during Step 2 (T2 to T3); *K-SADS* Kiddie-Sads-Present and Lifetime Version with the scales Inattention, Hyperactivity/Impulsivity and Oppositional defiant disorder, *SDQ* Strengths and Difficulties Questionnaire with the scales Hyperactivity, Conduct Problems and Emotional Symptoms, *HSQ* Home Situation Questionnaire, *FIQ* Family Impact Questionnaire with the scales Impact on Social Life, Positive Feelings Toward Child and Negative Feelings Toward Child

^*^p < .05

**Table 2:** Change in Mother Outcome Variables During Step 1 Treatment of the Mother and Step 2 Parent-Child Training for Standardized Variables

Outcome & informant	Control group		Treatment group	
	${\upalpha}_2^{\left(\mathrm{CG}\right)}$	${\upalpha}_3^{\left(\mathrm{CG}\right)}$	${\upalpha}_2^{\left(\mathrm{TG}\right)}$	${\upalpha}_3^{\left(\mathrm{TG}\right)}$
CAARS–O blinded clinician				
Inattention	−0.19^*^	− 0.19^*^	− 0.60^*^	− 0.19^*^
Hyperactivity	− 0.20^*^	− 0.20^*^	− 0.35^*^	− 0.35^*^
Impulsivity	− 0.25^*^	− 0.25^*^	− 0.62^*^	− 0.25^*^
CAARS–S mother				
Inattention	−0.18^*^	− 0.18^*^	− 0.58^*^	− 0.18^*^
Hyperactivity	− 0.24^*^	− 0.24^*^	− 0.55^*^	− 0.24^*^
Impulsivity	− 0.23^*^	− 0.23^*^	− 0.62^*^	− 0.23^*^

Note. Results concern the final model of a series of nested piecewise linear latent growth models (within- and between-group analysis). In the case of equal coefficients, change rates did not significantly differ across treatment groups and/or steps and were constrained to be equal. ${\upalpha}_2^{\left(\mathrm{CG}\right)}$ = mean of latent slope factor representing average change in CG during Step 1 (T1 to T2); ${\upalpha}_3^{\left(\mathrm{CG}\right)}$ = mean of latent slope factor indicating average change in CG during Step 2 (T2 to T3); ${\upalpha}_2^{\left(\mathrm{TG}\right)}$ = mean of latent slope factor representing average change in TG during Step 1 (T1 to T2); ${\upalpha}_3^{\left(\mathrm{TG}\right)}$ = mean of latent slope factor indicating average change in TG during Step 2 (T2 to T3); *CAARS–O* Conners’ Adult ADHD Rating Scales–Observer: Long Version with the scales Inattention and Memory Problems, Hyperactivity/Restlessness and Impulsivity/Emotional Lability, *CAARS–S* Conners’ Adult ADHD Rating Scales–Self-Report: Long Version with scales analogous to CAARS–O

^*^p < .05

Results for unstandardized variables of child and mother outcome are provided in Additional file 1: Table S2. This table also contains additional information about model fit (χ^2^ test, *CFI*) and the means of the latent intercept factor α_1_.

### Within-group analysis

In the within-group analysis, the equality of the Step 1 and Step 2 slope factor means was tested within the groups. This primarily served as a test for the additional effects of the Step 2 parent-child training in the TG and the CG after the Step 1 treatment of the mother. In the CG and for child outcome (Table 1), ${\upalpha}_2^{\left(\mathrm{CG}\right)}$ and ${\upalpha}_3^{\left(\mathrm{CG}\right)}$ did not significantly differ, except for child disruptive behavior rated by the blinded clinician (K-SADS ODD) and the mother (SDQ Conduct). For these two measures, a stronger symptom decrease was observed during the Step 2 parent-child training compared to the previous Step 1 treatment of the mother, indicating additional treatment effects of the parent-child training in the CG. For mother outcome in the CG (Table 2), no differences between ${\upalpha}_2^{\left(\mathrm{CG}\right)}$ and ${\upalpha}_3^{\left(\mathrm{CG}\right)}$ were detected.

Regarding the TG and child outcome (Table 1), no differences between ${\upalpha}_2^{\left(\mathrm{TG}\right)}$ and ${\upalpha}_3^{\left(\mathrm{TG}\right)}$ were found, and therefore no evidence for any additional effect of the parent-child training after the Step 1 intensive treatment of the mother. In contrast, varying change rates in the TG during Step 1 and Step 2 were obtained for ADHD symptoms of the mothers (Table 2) rated by the blinded clinician (CAARS–O Inattention, CAARS–O Impulsivity) and the mothers themselves (CAARS–S Inattention, CAARS–S Hyperactivity, CAARS–S Impulsivity). Both informants reported a stronger symptom reduction during the Step 1 treatment of the mother compared to the Step 2 parent-child training.

### Between-group analysis

This analysis considered two comparisons: First, the contrast of the Step 1 slope factor means of the CG and TG (H_0_: ${\upalpha}_2^{\left(\mathrm{CG}\right)}$ = ${\upalpha}_2^{\left(\mathrm{TG}\right)}$) and second, the comparison of the Step 2 factor means of both groups (H_0_: ${\upalpha}_3^{\left(\mathrm{CG}\right)}$ = ${\upalpha}_3^{\left(\mathrm{TG}\right)}$). The Step 1 comparison was of particular interest for this analysis, as it served as a test for the efficacy of the intensive treatment of the mother in the TG relative to the CG. For child outcome (Table 1), differences between groups were detected only for disruptive behavior during Step 1 in ratings of the mothers (SDQ Conduct) and during Step 2 in ratings of the blinded clinicians (K-SADS ODD). More specifically, throughout Step 1, mothers of the CG reported no symptom change, while mothers of the TG rated a reduction (SDQ Conduct). This indicated the efficacy of the intensive treatment of the mother in the TG relative to the CG regarding the child’s disruptive behavior. In ratings of the blinded clinicians, group differences emerged at Step 2 during parent-child training and were in favor of the CG in terms of a stronger reduction of disruptive behavior (K-SADS ODD).

With respect to ADHD symptoms of mothers (Table 2) during Step 1, there was a consistent treatment advantage for the TG in ratings of the blinded clinicians (CAARS–O Inattention, CAARS-O Hyperactivity, CAARS–O Impulsivity) as well as in reports of the mothers (CAARS–S Inattention, CAARS-S Hyperactivity, CAARS–S Impulsivity). Subsequently, during Step 2, the TG benefit was only assessed for hyperactivity reported by the blinded clinicians (CAARS-O Hyperactivity).

### Effect size and informants

Factor means (${\upalpha}_2^{\left(\mathrm{CG}\right)}$, ${\upalpha}_3^{\left(\mathrm{CG}\right)}$, ${\upalpha}_2^{\left(\mathrm{TG}\right)}$, ${\upalpha}_3^{\left(\mathrm{TG}\right)}\Big)$ of the standardized observed variables (Tables 1 and 2) were considered as effect size measures and were interpreted like Cohen’s *d* (see Statistical analysis section). The effect sizes varied depending on different factors including the outcome measure, the informant, and the treatment period. For child symptoms (Table 1), on a descriptive level, the strongest change was observed for disruptive behavior in the CG during Step 2 as rated by the blinded clinician, with an effect size in the medium range (K-SADS ODD). In contrast, as expected, teachers reported comparatively low effect sizes for child symptoms in general (SDQ). For ADHD symptoms of the mother (Table 2), the most pronounced change was detected in the TG during Step 1 treatment of the mother rated by the blinded clinicians (CAARS–O) as well as the mothers (CAARS–S), with effect sizes in the medium range.

### Model fit and intercept factor

Model fit criteria were not fulfilled for all outcome variables in multi-group analysis (Additional file 1: Table S2). Therefore, for each variable, we inspected which group (CG, TG) was the source for insufficient model fit and did not achieve model fit criteria when analyzed in single-group analysis. However, we refrained from any post-hoc model specifications because the consideration of correlated residuals often resulted in model errors.

The between-group analysis also included a comparison of the intercept factor means α_1_ that indicated the average outcome at baseline (Additional file 1: Table S2). Except for one scale in mother rating (FIQ Social), no group differences were detected between the CG and TG.

## Discussion

The AIMAC study aimed to test, in a randomized trial, whether an intensive treatment for mothers with ADHD including pharmacotherapy and group psychotherapy, improves outcomes for ADHD parent-child training. Previous analyses showed that ADHD symptoms could be successfully reduced in mothers with intensive Step 1 treatment compared to minimal treatment. However, the combination of intensive treatment of the mother and subsequent parent-child training revealed no treatment advantage regarding ADHD symptoms and disruptive behavior of the child [32].

This secondary analysis was particularly concerned with the efficacy of the parent-child training in the AIMAC study. The results of our analysis suggest that the parent-child training may be a valid treatment option and may help to reduce the disruptive behavior (but not ADHD symptoms) of the child even for mothers who are not intensively treated beforehand. This can be concluded from the within-group analysis of the CG in reports of the blinded clinician and the mothers (CG: Step 1 vs. Step 2). The results are of practical importance as they suggest that children’s disruptive behavior may improve from parent-child training even if the mothers still show ADHD symptoms and do not receive the best available treatment in advance. Previous research findings on this topic have been mixed [5, 6]. As suggested in previous reviews, the positive results in this trial may be explained by the personalized approach we used in order to accommodate the training to the needs of the parents [5]. The individualized treatment planning may have helped the mothers to compensate for ADHD-related deficits and to participate in the training.

The efficacy of the parent-child training in the CG may also contribute to the understanding of why, in previous studies, the two treatment groups may not have differed when the total change across both treatment steps was analyzed [32]. Our results suggest that what the CG may have missed out on in terms of improvement during the first phase of the study (due to the lower intensity of the treatment of the mother), was likely made up for during the second phase when the parent-child training was offered. For the TG, by contrast, there is a tendency, across informants, that the improvement of the disruptive behavior already began earlier, during the treatment of the mother, and was more equally distributed over the two treatment steps. TG families might therefore have had less room for improvement at the beginning of the parent-child training. Furthermore, although we found no evidence for an additional effect of the parent-child training in the TG, we would not conclude that this treatment approach was less relevant for these children. The parent-child training might have been important to maintain the initial Step 1 treatment gains, and the limitations of our analytical approach have to be considered (see limitations section).

The second research question concerned the efficacy of the intensive treatment of the mother (pharmacotherapy, psychotherapy) regarding child symptoms. Compared to supportive counseling in the CG, children of the TG showed a treatment advantage regarding disruptive behavior in the ratings of the mothers at the end of the first treatment phase (Step 1: TG vs. CG). To our knowledge, this is the first study to demonstrate a treatment advantage of the combined effect of adult pharmacotherapy and psychotherapy on the child outcome. We consider this finding to be robust, as we tested against an active control condition in which at least some common treatment factors were realized (e.g., hope, therapeutic alliance). One might speculate further that the effect of the adult treatment in the TG on the disruptive behavior of the child was mediated by improved parenting practices, which are considered to be the link between neuropsychological functioning of the parent and the psychopathology of the child [11]. However, the effect of the adult treatment on the child was rather small.

Positive effects for child disruptive behavior were observed in ratings of the blinded clinician and the mother but not in teacher ratings. The latter may be explained by a lack of generalization of the improvement to different settings. This is a common finding and has been reported in other studies as well [58]. Although we consider the blinded clinician perspective to be valuable, restrictions also need to be considered here. As clinical ratings were largely based on a parent interview, their assessment might have been biased towards the perspective of the mother [32].

Of further interest is our observation of a reduction of child ADHD symptoms in both treatment groups. For each treatment group and step, the improvement was in the small to medium range for ratings of the blinded clinician and the mothers. For ratings of the teachers, the effects were negligible. However, we could not highlight any group or phase to be superior to the other (within- and between-group comparison). As a consequence, the internal validity of the findings is low and the results remain somewhat inconclusive. It might be that all interventions are effective, but it is not possible to estimate their treatment advantage compared to merely waiting, and other explanations for the symptom reductions are possible as well (e.g., regression to the mean).

One potentially important factor in explaining these results for child ADHD symptoms is child pharmacotherapy which already existed before the trial. Three quarters of the children were maintained on medication throughout the study. As indicated by rather low baseline values, this probably reduced the room for improvement in child ADHD symptoms and consequently the likelihood of detecting treatment effects. For many children, the results therefore rather reflect the additional effect of study treatments beyond that of an existing medication. However, in recent moderator analyses, ongoing child medication was found to have no explanatory power [59].

This study has several limitations. First, the analysis relied in part on a within-group control design, which is less optimal for drawing firm conclusions than the randomized between-group comparison. To test the additional treatment effect of the parent-child training, the change rates of the two treatment phases were contrasted. This test is based implicitly on the assumption that the change during the first step is maintained in the second step, which in our case was a very conservative assumption particularly for the efficacy of the parent-child training in the TG. Second, model fit criteria were quite liberal and, moreover, were not reached in all outcome variables. Multiple factors may be responsible for misfit in growth curve models [60, 61], including misspecifications in the mean structures (e.g., functional form of the mean growth trajectory) and the covariance structures (e.g., covariance of residuals). As change during Step 1 and Step 2 was determined by two assessment points only, we could not investigate other growth forms besides the linear models. We also abstained from any post-hoc model modifications regarding the covariance structure (e.g., adding correlated residuals), because the consideration of correlated residuals often resulted in model errors. Furthermore, we were primarily interested in the mean growth trajectory, which is less affected by possible misspecifications in the covariance structure [60]. Third, our results do not always completely correspond to previous findings of the study (e.g., SDQ Conduct) [32]. Among other things, this is due to differences in the research question and varying statistical models. In Jans et al. [32] the focus was on the between-group differences (CG vs. TG). For this a linear regression approach was used and the outcome at certain time points was predicted by the treatment group along with other covariates including the baseline assessment. In contrast for the current analysis we also were interested in the within-group perspective (Step 1 vs. Step 2 in CG and TG). For this change scores (T1 to T2; T2 to T3) were estimated by piecewise latent growth curve models that could be contrasted not only between but also within the treatment groups. A detailed discussion about other and more general concerns regarding this study has been provided elsewhere [32, 62].

## Conclusions

We conclude from our findings that parent-child training may be effective to reduce children’s disruptive behavior even if parental ADHD is not intensively treated beforehand. This is in contrast to some previous findings. The discrepant results may be explained by different methodological approaches, although we hypothesize that the positive effects in this analysis were mainly attributable to the fact that the parent-child training was individualized, which may have helped the parents to compensate for ADHD-related deficits and to enhance their treatment participation. Future studies should investigate the importance of personalized treatment approaches for parents with ADHD in more detail and under experimental control. We also found that the combination of pharmacotherapy and psychotherapy for mothers improved children’s disruptive behavior, at least from the perspective of the participating mothers. However, the effects were rather small and may not be sufficient to fully support the child. This finding is in line with previous studies investigating the effect of adult pharmacotherapy on children in isolation. The results of our analysis add to the existing knowledge that even a multimodal approach for ADHD in parents might not be sufficient for their children. Therefore, if both parent and child are diagnosed with ADHD, a child-centered approach is recommended in any case.

## Additional file

Additional file 1: This article is accompanied by an online supplement containing additional information regarding the analysis. (PDF 376 kb)

## Abbreviations

ADHD: Attention-deficit/hyperactivity disorder
AIMAC: ADHD in Mothers and Children; study under investigation
CAARS–L: Conners’ Adult ADHD Rating Scales–Self-Report: Long Version; questionnaire rated by mothers
CAARS–O: Conners’ Adult ADHD Rating Scales–Observer: Long Version; questionnaire rated by blinded clinicians
CFI: Comparative fit index
CG: Control group; receives supportive counseling during Step 1 and parent-child training in Step 2
DSM-IV: Diagnostic and Statistical Manual of Mental Disorders (4th ed.)
FAS: Full analysis set; sample that considers only families with the primary outcome available at baseline
FIQ: Family Impact Questionnaire; questionnaire rated by mothers
H_0_: Null hypothesis
HSQ: Home Situation Questionnaire; questionnaire rated by mothers
K-SADS: Kiddie-Sads-Present and Lifetime Version; semi-structured interview rated by blinded clinician
ODD: Oppositional defiant disorder
RMSEA: Root mean square error of approximation
SDQ: Strengths and Difficulties Questionnaire; questionnaire rated by mothers and teachers
Step 1: First treatment phase of 3 months; encompasses treatment of the mother
Step 2: Second treatment phase of 3 months; encompasses parent-child training and continued treatment of the mother
Step 3: Third treatment phase of 3 months; encompasses maintenance period for the mother and the child treatment
T1: Assessment at baseline
T2: Assessment immediately after Step 1 about 3 months after T1
T3: Assessment immediately after Step 2 about 6 months after T1
T4: Assessment immediately after Step 3 about 12 months after T1
T5: Follow-up assessment approximately 1 year after Step 3
TG: Treatment group; receives group psychotherapy and stimulant medication during Step 1 and parent-child training in Step 2
THOP: Therapieprogramm für Kinder mit hyperkinetischem und oppositionellem Problemverhalten (Therapy program for children with hyperkinetic and oppositional problem behavior); a German treatment manual
WURS-K: Wender Utah Rating Scale, German short version, questionnaire rated by mothers
