# Supplementary material for: Sequential treatment of ADHD in mother and child (AIMAC study): importance of the treatment phases for intervention success in a randomized trial
Source: BMC Psychiatry. 2018 Dec 13;18:388. doi: 10.1186/s12888-018-1963-9 (PMC6293507; doi:10.1186/s12888-018-1963-9)
Supplement: Supplementary file 1 — This article is accompanied by an online supplement containing additional information regarding the analysis. (PDF 376 kb) [file 12888_2018_1963_MOESM1_ESM.pdf]

Supplement

Sequential treatment of ADHD in mother and child (AIMAC study): Importance of the treatment phases for intervention success in a randomized trial

This supplement contains supporting information for the above-mentioned manuscript.

Figure S1. Study design of the AIMAC study (ADHD in Mothers and Children) including treatment of the mother and parent-child training for treatment group and control group families 2
Figure S2. Multi-group analysis of piecewise latent growth curve models 3

Table S1. Descriptive statistics of outcome measures before and after Step 1 (T1 to T2) mother treatment and Step 2 (T2 to T3) parent-child training...4
Table S2. Results of the final multi-group analysis of piecewise linear latent growth models for unstandardized variables 6

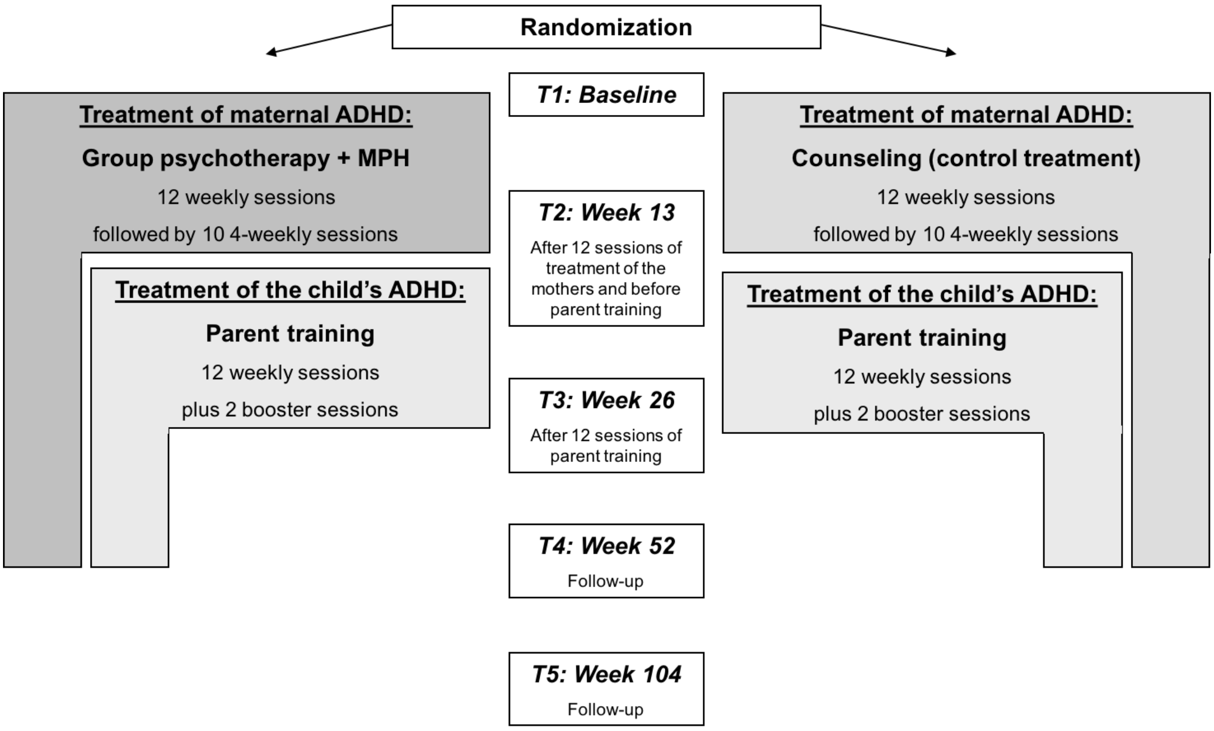

Figure S1. Study design of the AIMAC study (ADHD in Mothers and Children) including treatment of the mother and parent-child training for treatment group and control group families. Adapted from "Does the treatment of maternal attention deficit and hyperactivity disorder (ADHD) enhance the efficacy of a behavioural parent training for the treatment of their children's ADHD? Study protocol of a randomized controlled multicentre trial," by T. Jans, A. Philipsen, E. Graf, G. Ihorst, M. Gerlach, and A. Warnke. 2009, *Attention Deficit and Hyperactivity Disorders, 1*, p. 36. Copyright by Springer Vienna.

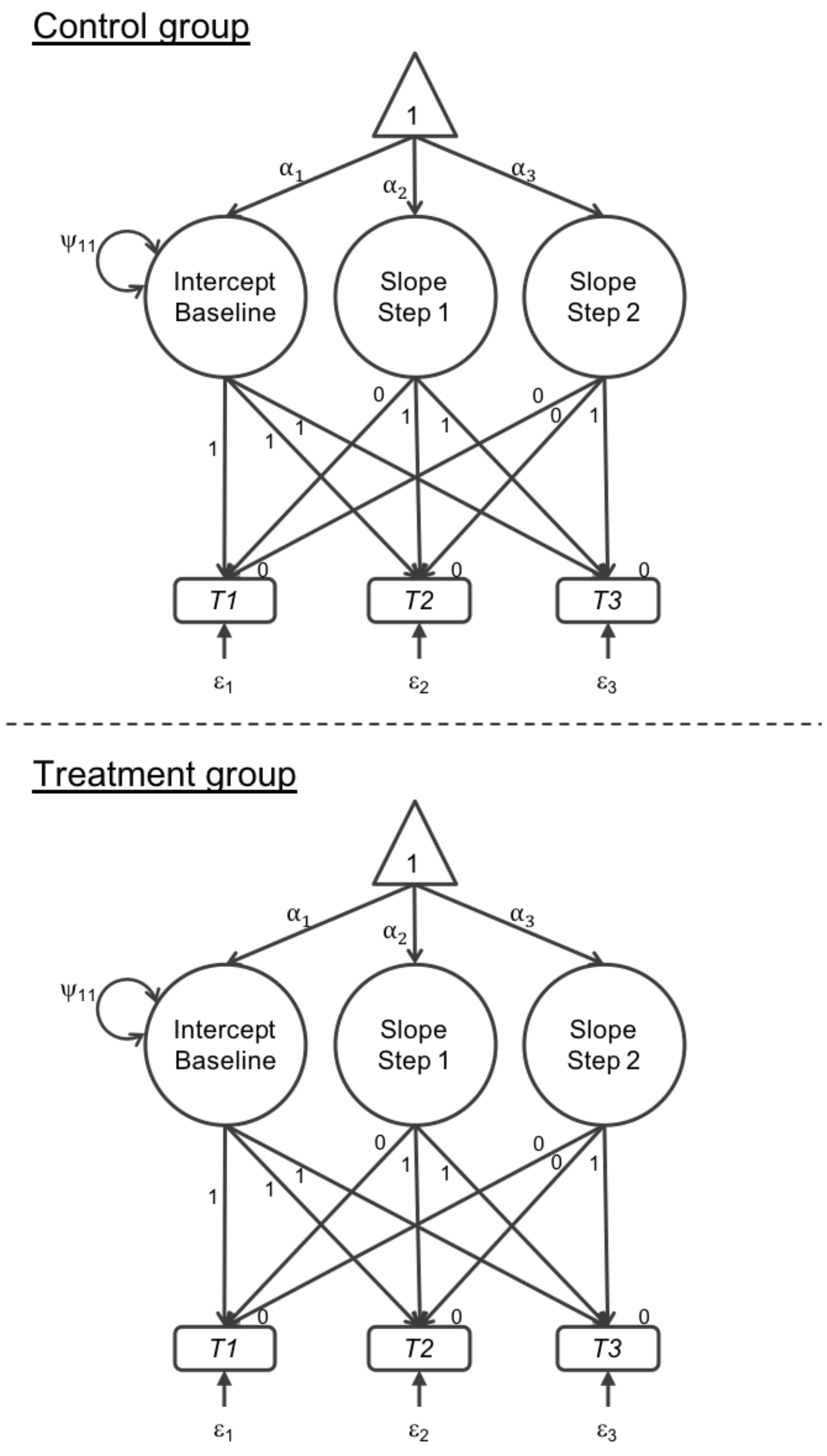

Figure S2. Multi-group analysis of piecewise latent growth curve models. ψ_{11} = variance latent intercept factor representing interindividual variation at baseline; α_1 = mean latent intercept factor indicating average value at baseline; α_2 = mean latent slope factor for Step 1 representing average change during treatment of the mother; α_3 = mean latent slope factor for Step 2 indicating average change during parent-child training; T1 = baseline assessment; T2 = assessment after Step 1 mother treatment and before Step 2 parent-child training; T3 = assessment after Step 2 parent-child training; ε_1–ε_3 = error terms of the respective assessment points.

Table S1. Descriptive statistics of outcome measures before and after Step 1 (T1 to T2) mother treatment and Step 2 (T2 to T3) parent-child training

	Control group							Treatment group						
		T1		T2		T3			T1		T2		T3	
Outcome & informant	*n*	*M*	(*SD*)	*M*	(*SD*)	*M*	(*SD*)	*n*	*M*	(*SD*)	*M*	(*SD*)	*M*	(*SD*)
Child outcome														
K-SADS blinded clinician														
Inattention	66	5.89	(1.97)	4.72	(2.03)	3.76	(2.17)	77	5.86	(2.45)	4.78	(2.35)	3.93	(2.49)
Hyp/Imp	66	5.30	(2.41)	4.76	(2.22)	3.77	(2.16)	77	5.25	(2.45)	5.08	(2.42)	4.25	(2.67)
ODD	66	3.70	(1.95)	3.60	(2.01)	2.33	(1.92)	77	3.65	(2.15)	3.32	(2.16)	2.93	(2.01)
SDQ mother														
Hyperactivity	66	7.20	(1.94)	6.63	(1.93)	6.21	(2.01)	77	7.24	(1.77)	6.61	(1.98)	5.92	(2.43)
Conduct	66	3.80	(2.10)	3.97	(2.01)	3.27	(1.75)	77	4.31	(2.06)	3.91	(1.66)	3.38	(1.96)
Emotional	66	3.73	(2.51)	3.57	(2.34)	2.72	(2.18)	77	4.04	(2.19)	4.00	(2.25)	3.26	(2.18)
HSQ mother														
Total	66	50.19	(26.20)	46.54	(25.98)	37.64	(23.62)	77	56.21	(25.87)	51.32	(27.22)	40.44	(23.01)
FIQ mother														
Social	66	0.93	(0.55)	0.88	(0.52)	0.80	(0.56)	77	1.15	(0.60)	1.04	(0.65)	0.92	(0.57)
Negative	65	1.54	(0.54)	1.47	(0.54)	1.27	(0.55)	76	1.50	(0.51)	1.47	(0.58)	1.27	(0.57)
Positive	65	0.98	(0.58)	0.95	(0.60)	0.97	(0.57)	76	0.98	(0.55)	1.04	(0.68)	1.08	(0.63)
SDQ teacher														
Hyperactivity	57	5.05	(2.74)	4.53	(2.46)	4.46	(2.32)	65	5.03	(2.93)	4.49	(2.41)	4.52	(2.82)
Conduct	57	2.12	(2.27)	1.93	(1.94)	1.61	(1.66)	65	2.51	(2.38)	1.99	(1.69)	1.47	(1.48)
Emotional	57	2.22	(2.46)	2.00	(2.30)	1.45	(1.81)	65	2.48	(2.27)	2.39	(1.89)	2.22	(1.86)
Mother outcome														
CAARS–O blinded clinician														
Inattention	66	17.57	(7.19)	16.31	(7.40)	15.19	(8.32)	76	18.29	(7.00)	13.87	(7.53)	11.86	(6.84)
Hyperactivity	66	17.12	(7.82)	15.00	(7.29)	14.12	(6.68)	76	16.68	(6.06)	13.26	(7.11)	11.67	(6.53)
Impulsivity	66	19.26	(7.33)	16.68	(6.49)	15.28	(7.50)	76	18.23	(7.51)	13.85	(6.11)	11.97	(6.16)
CAARS–S mother														
Inattention	65	17.36	(7.85)	15.87	(8.31)	14.96	(8.41)	75	18.44	(7.34)	13.88	(7.27)	11.98	(6.65)
Hyperactivity	65	16.37	(7.05)	14.15	(6.60)	13.06	(6.08)	75	16.17	(6.64)	12.28	(6.42)	10.80	(6.02)
Impulsivity	65	19.26	(7.49)	17.40	(7.63)	15.52	(8.22)	75	18.63	(7.74)	13.89	(6.49)	12.17	(6.48)

Note. Missing data were handled by FIML. For a person to be considered for analysis, at least one assessment per variable was required. *n* = available sample size for analysis; T1 = baseline assessment; T2 = assessment after Step 1 mother treatment and before Step 2 parent-child training; T3 = assessment after Step 2 parent-child training; K-SADS = Kiddie-Sads-Present and Lifetime Version with the scales Inattention, Hyperactivity/Impulsivity and Oppositional Defiant Disorder; SDQ = Strengths and Difficulties Questionnaire with the scales Hyperactivity, Conduct Problems and Emotional Symptoms; HSQ = Home Situation Questionnaire; FIQ = Family Impact Questionnaire with the scales Impact on Social Life, Positive Feelings Toward Child and Negative Feelings Toward Child; CAARS–O = Conners' Adult ADHD Rating Scales–Observer:

Long Version with the scales Inattention and Memory Problems, Hyperactivity/Restlessness and Impulsivity/Emotional Lability; CAARS–S = Conners' Adult ADHD Rating Scales–Self Report: Long Version with scales analogous to CAARS–O.

Table S2. Results of the final multi-group analysis of piecewise linear latent growth models for unstandardized variables

					Control group			Treatment group			
Outcome & informant	*df*	χ^2	*p*	*CFI*	$\alpha_1^{(CG)}$	$\alpha_2^{(CG)}$	$\alpha_3^{(CG)}$	$\alpha_1^{(TG)}$	$\alpha_2^{(TG)}$	$\alpha_3^{(TG)}$	Source of misfit
					Child outcome						
K-SADS blinded clinician											
Inattention	8	1.29	1.00	1.00	5.85*	–1.03*	–1.03*	5.85*	–1.03*	–1.03*	
Hyp/Imp	8	8.64	.37	.99	5.36*	–0.62*	–0.62*	5.36*	–0.62*	–0.62*	
ODD	7	4.80	.68	1.00	3.72*	–0.34*	–1.11*	3.72*	–0.34*	–0.34*	
SDQ mother											
Hyperactivity	8	12.29	.14	.96	7.23*	–0.61*	–0.61*	7.23*	–0.61*	–0.61*	TG
Conduct	7	17.40	.01	.93	4.15*	–0.10	–0.47*	4.15*	–0.47*	–0.47*	TG
Emotional	8	16.60	.03	.93	3.99*	–0.44*	–0.44*	3.99*	–0.44*	–0.44*	CG
HSQ mother											
Total	8	13.80	.09	.95	54.42*	–7.03*	–7.03*	54.42*	–7.03*	–7.03*	
FIQ mother											
Social	7	7.81	.35	1.00	0.95*	–0.09*	–0.09*	1.13*	–0.09*	–0.09*	
Negative	8	10.59	.23	.98	1.53*	–0.11*	–0.11*	1.53*	–0.11*	–0.11*	
Positive	8	6.34	.61	1.00	0.98*	0.02	0.02	0.98*	0.02	0.02	
SDQ teacher											
Hyperactivity	8	4.94	.76	1.00	4.95*	–0.30*	–0.30*	4.95*	–0.30*	–0.30*	
Conduct	8	15.56	.05	.89	2.29*	–0.34*	–0.34*	2.29*	–0.34*	–0.34*	TG
Emotional	8	5.60	.69	1.00	2.47*	–0.29*	–0.29*	2.47*	–0.29*	–0.29*	
					Mother outcome						
CAARS–O blinded clinician											
Inattention	7	14.34	.05	.96	18.05*	–1.51*	–1.51*	18.05*	–4.63*	–1.51*	TG
Hyperactivity	7	25.13	.00	.88	16.54*	–1.48*	–1.48*	16.54*	–2.51*	–2.51*	TG
Impulsivity	7	6.07	.53	1.00	18.58*	–1.86*	–1.86*	18.58*	–4.60*	–1.86*	
CAARS–S mother											
Inattention	7	5.48	.60	1.00	17.99*	–1.46*	–1.46*	17.99*	–4.61*	–1.46*	
Hyperactivity	7	7.54	.37	1.00	16.15*	–1.66*	–1.66*	16.15*	–3.81*	–1.66*	
Impulsivity	7	2.00	.96	1.00	18.92*	–1.79*	–1.79*	18.92*	–4.86*	–1.79*	

Note. Results concern the final model of a series of nested piecewise linear latent growth models (within- and between-group analysis). *df* = degrees of freedom; χ^2 = chi-square value; *CFI* = comparative fit index; $\alpha_1^{(CG)}$ = mean of latent intercept factor indicating average outcome in CG at baseline (T1); $\alpha_2^{(CG)}$ = mean of latent slope factor representing average change in CG during Step 1 (T1 to T2); $\alpha_3^{(CG)}$ = mean of latent slope factor indicating average change in CG during Step 2

(T2 to T3); $\alpha_1^{(TG)}$ = mean of latent intercept factor indicating average outcome in TG at baseline (T1); $\alpha_2^{(TG)}$ = mean of latent slope factor representing average change in TG during Step 1 (T1 to T2); $\alpha_3^{(TG)}$ = mean of latent slope factor indicating average change in TG during Step 2 (T2 to T3); misfit = indicates intervention group (CG, TG) with one or both criteria of model fit not fulfilled (χ^2 test $p > .05$; $CFI > .90$) in single-group analysis (within-group analysis); K-SADS = Kiddie-Sads-Present and Lifetime Version with the scales Inattention, Hyperactivity/Impulsivity and Oppositional Defiant Disorder; SDQ = Strengths and Difficulties Questionnaire with the scales Hyperactivity, Conduct Problems and Emotional Symptoms; HSQ = Home Situation Questionnaire; FIQ = Family Impact Questionnaire with the scales Impact on Social Life, Positive Feelings Toward Child and Negative Feelings Toward Child; CAARS–O = Conners' Adult ADHD Rating Scales–Observer: Long Version with the scales Inattention and Memory Problems, Hyperactivity/Restlessness and Impulsivity/Emotional Lability; CAARS–S = Conners' Adult ADHD Rating Scales–Self Report: Long Version with scales analogous to CAARS–O; TG = treatment group; CG = control group.
* $p < .05$.
